# Glycated albumin is more closely correlated with coronary artery disease than 1,5-anhydroglucitol and glycated hemoglobin A1c

**DOI:** 10.1186/s12933-014-0166-z

**Published:** 2015-02-07

**Authors:** Xiaojing Ma, Xiang Hu, Jian Zhou, Yaping Hao, Yuqi Luo, Zhigang Lu, Yuqian Bao, Weiping Jia

**Affiliations:** Department of Endocrinology and Metabolism, Shanghai Jiao Tong University Affiliated Sixth People’s Hospital, Shanghai Clinical Center for Diabetes, Shanghai Key Clinical Center for Metabolic Disease, Shanghai Diabetes Institute; Shanghai Key Laboratory of Diabetes Mellitus, 600 Yishan Road, Shanghai, 200233 China; Department of Cardiology, Shanghai Jiao Tong University Affiliated Sixth People’s Hospital, Shanghai, 200233 China

**Keywords:** Coronary artery disease, Coronary angiography, Glycated albumin, Glycated hemoglobin A1c, 1,5-anhydroglucitol

## Abstract

**Background:**

The aim of this study was to investigate the associations of two nontraditional glycemic markers, glycated albumin (GA) and 1,5-anhydroglucitol (1,5-AG), as well as glycated hemoglobin A1c (HbA1c) with coronary artery disease (CAD).

**Methods:**

In total, 272 subjects (178 men and 94 postmenopausal women) were enrolled in this study. All of them underwent coronary angiography which was used to diagnose CAD. The severity of coronary artery stenosis was assessed by the coronary stenosis index (CSI). GA and 1,5-AG were assayed using the enzymatic method, and HbA1c was detected by high-pressure liquid chromatography.

**Results:**

The HbA1c and GA levels were significantly higher in CAD group than those in non-CAD group (both *P* < 0.01). While the 1,5-AG level was significantly lower in CAD group than that in non-CAD group (*P* < 0.05). After adjustment for traditional risk factors of CAD, HbA1c, 1,5-AG, and GA, multivariate logistic regression analysis showed that GA was an independent risk factor for CAD (odds ratio = 1.143, 95% confidence interval: 1.048-1.247, *P* = 0.002). With CSI as a dependent variable, multiple stepwise regression analysis demonstrated an independent positive correlation between GA and CSI (standardized *β* = 0.184, *P* = 0.003), beyond gender, age, and lipid-lowering therapy, after adjustment for traditional risk factors of CAD, HbA1c, 1,5-AG, and GA.

**Conclusions:**

GA was more closely correlated with CAD than HbA1c and 1,5-AG in a Chinese population with high risk of CAD.

## Background

Diabetes mellitus is a coronary artery disease (CAD) risk equivalent. Abnormalities in glucose metabolism give rise to an increased incidence of CAD, and blood glucose level is considered to be a continuous risk factor for cardiovascular disease [1]. Clinical studies have revealed that atherosclerosis, a chronic inflammatory process, is activated not after the diagnosis of diabetes, but rather in the early stage of hyperglycemia, even when the glycemic level is not high enough to indicate the presence of diabetes. Furthermore, the process of atherosclerosis is accelerated in patients with elevated blood glucose level [2]. Thus, research exploring on the correlations of standard glycemic marker [*e.g.*, glycated hemoglobin A1c (HbA1c)] and some nontraditional glycemic markers [*e.g.*, glycated albumin (GA), which reflects the blood glucose level during the previous 2 to 3 weeks, and 1,5-anhydroglucitol (1,5-AG), which reflects the blood glucose level during the previous 1 to 2 weeks] with CAD to identify which glycemic markers are more closely related to CAD is of clinical importance for early screening and intervention of CAD.

A series of studies conducted among Chinese patients with type 2 diabetes mellitus suggested that GA was superior to HbA1c for identifying the presence and severity of CAD [3-5]. Additionally, Ikeda, et al. [6] found that the serum 1,5-AG level was more valuable than the HbA1c level as a predictor of CAD in Japanese patients. To date, however, few studies have simultaneously compared the relationships of the presence of CAD between HbA1c, GA, and 1,5-AG levels in Chinese population. In the present study, we used coronary angiography to diagnose CAD and calculated the coronary stenosis index (CSI) to assess the severity of coronary artery stenosis [7]. The goal of this study was to investigate the associations of HbA1c, GA, and 1,5-AG with the presence and severity of CAD and to identify a suitable indicator for early screening and prevention of CAD.

## Methods

### Subjects

The subjects in this study were recruited from the Department of Cardiology of Shanghai Jiao Tong University Affiliated Sixth People’s Hospital from July 2008 to January 2010. All subjects underwent coronary angiography and provided complete clinical data. We excluded subjects with the following factors affecting HbA1c, GA, and 1,5-AG measurements and analyses: myocardial infarction within 3 months, history of coronary artery bypass grafting or percutaneous coronary intervention within 6 months; congestive heart failure; acute infection; psychosis; autoimmune disease; hematopathy; liver or renal dysfunction; hyperthyroidism or hypothyroidism; history of malignancy, severe trauma or gastrectomy; or use of traditional Chinese medicine.

All subjects completed a standard questionnaire about their disease history, medication history, family history of CAD and smoking status along with the complete results of their physical examination, blood pressure measurement, and parameters of glucose and lipid metabolism. A current smoking status was defined as regular smoking or smoking at least one cigarette per day for more than 6 months [8]. Finally, 272 subjects (age range, 38-86 yr, mean 65.7 ± 10.2 yr), including 178 men and 94 postmenopausal women, were enrolled in this study. The diagnosis of diabetes was established according to the criteria proposed by the World Health Organization in 1999 [9]. This study was approved by the local Ethics Committee of Shanghai Jiao Tong University Affiliated Sixth People’s Hospital, and all subjects provided written informed consent.

### Anthropometric measurements

The body mass index (BMI) of each subject was calculated as weight in kilograms divided by the square of height in meters. Waist circumference (W) was measured in the standing position midway between the lowest rib and the iliac crest. Blood pressure was detected with a mercury sphygmomanometer after the subjects had rested for 10 minutes.

### Laboratory measurements

Blood samples of all subjects were obtained in the morning after a 10-hour overnight fast. A 75-g oral glucose tolerance test was performed among subjects without history of diabetes, while a 100-g carbohydrate test was carried out for the known diabetic patients. The fasting plasma glucose level (FPG) and 2-hour postprandial plasma glucose level (2hPG) were assessed by the glucose oxidase method. The serum creatinine level (Scr) and lipid profiles including the triglyceride level (TG), total cholesterol level (TC), low-density lipoprotein cholesterol level (LDL-c), and high-density lipoprotein cholesterol level (HDL-c) were quantified by enzymatic methods on a 7600-020 autoanalyzer (Hitachi, Tokyo, Japan). The serum C-reactive protein level (CRP) was determined via particle-enhanced immunonephelometry assay (Dade Behring Inc., Newark, NJ, USA). The fasting serum insulin level (FINS) was determined via radioimmunoassay (Linco Research, St. Charles, MO, USA) and was applied to calculate the homeostasis model assessment-insulin resistance index (HOMA-IR) as follows: HOMA-IR = FPG (mmol/L) × FINS (mU/L)/ 22.5 [10]. The estimated glomerular filtration rate (eGFR, mL/min/1.73 m^2^) was calculated according to the following equation recommended by the Modification of Diet in Renal Disease study: eGFR = [186 × (Scr/88.4) ^-1.154^ × (age) ^-0.203^ × 0.742 (if women)] [11].

HbA1c was detected by high-pressure liquid chromatography (Variant II hemoglobin analyzer; Bio-Rad, Hercules, CA, USA) with inter- and intra-assay coefficients of variation of < 3.40% and < 2.60%, respectively. GA was measured via a liquid enzymatic method (Lucica GA-L; Asahi Kasei Pharma Corporation, Tokyo, Japan) on a Glamour 2000 autoanalyzer (Molecular Devices, Sunnyvale, CA, USA) with inter- and intra-assay coefficients of variation of < 5.10% and < 3.00%, respectively. Finally, serum 1,5-AG was assayed by an enzymatic method (GlycoMark; GlycoMark Inc., New York, NY, USA) [12] on a 7600-120 autoanalyzer (Hitachi, Tokyo, Japan) with inter- and intra-assay coefficients of variation of < 2.19% and < 1.54%, respectively.

### Diagnosis of CAD

Coronary angiography was performed according to the method of Judkins [13]. CAD was defined as the presence of a luminal diameter stenosis of ≥ 50% in the left main coronary artery, left anterior descending artery or its first diagonal branch, left circumflex artery or its first obtuse marginal branch, and right coronary artery. Two experienced cardiologists performed coronary angiography on every participant and analyzed the images separately. The severity of coronary artery stenosis was assessed by calculating the CSI; the severity of coronary artery stenosis increased in parallel with the elevation of the CSI [7].

### Statistical analysis

SPSS, version 16.0 was used for statistical analysis. All variables underwent a normality test. Normally distributed data were expressed as the mean ± standard deviation, while skewed data were expressed as the median with interquartile range. For normally distributed data, intergroup comparisons were carried out using the unpaired Student’s *t* test; for skewed data, intergroup comparisons were conducted using the Wilcoxon rank sum test. Intergroup comparisons of categorical variables were carried out by the chi-squared test. Spearman correlation analysis and partial correlation analysis were conducted to explore the relationships among different glycemic indicators. Multivariate logistic regression analysis and multiple stepwise regression analysis were performed to identify the independent factors influencing the presence and severity of CAD. All the variables, which were traditional risk factors for CAD, as well as the disease-related therapies, were selected to enter into the regression analysis. All reported *P* values were two-tailed, and *P* < 0.05 was considered statistically significant.

## Results

### Clinical characteristics of the study participants

Of all 272 subjects, 194 (71.3%) were diagnosed with CAD. As shown in Table 1, compared with non-CAD group, subjects of CAD group were older and had higher levels of 2hPG, HbA1c, GA, and CSI (all *P* < 0.05), along with lower levels of 1,5-AG and TC (both *P* < 0.05), while FPG did not differ significantly between the two groups (*P >* 0.05). Subjects with CAD also exhibited a higher ratio of diabetes, use of hypoglycemic agents, and use of lipid-lowering drugs than subjects without CAD (all *P* < 0.05).

**Table 1:** **Population characteristics according to the presence or absence of CAD**

**Characteristics**	**Total**	**Non-CAD**	**CAD**
	**(N = 272)**	**(N = 78)**	**(N = 194)**
Gender (men/women)	178/94	39/39	139/55^aa^
Age (years)	65.7 ± 10.2	62.4 ± 10.0	67.0 ± 10.1^aa^
BMI (kg/m^2^)	24.8 ± 3.1	25.2 ± 3.2	24.6 ± 3.1
W (cm)	90.4 ± 9.2	90.1 ± 9.4	90.5 ± 9.1
SBP (mmHg)	130 (120-148)	130 (120-143)	130 (120-150)
DBP (mmHg)	80 (70-85)	80 (70-88)	80 (70-83)
FPG (mmol/L)	5.4 (5.0-6.3)	5.5 (5.1-6.0)	5.4 (5.0-6.5)
2hPG (mmol/L)	8.6 (6.7-11.8)	8.1 (6.0-9.9)	8.8 (6.7-12.5)^a^
HbA1c (%)	6.2 (5.7-6.8)	5.9 (5.6-6.4)	6.2 (5.8-7.0)^aa^
GA (%)	16.0 (14.0-19.0)	15.0 (13.0-17.0)	16.0 (15.0-19.0)^aa^
1,5-AG (μg/mL)	21.7 ± 10.1	23.9 ± 9.8	20.8 ± 10.1^a^
TC (mmol/L)	4.4 ± 1.1	4.6 ± 1.0	4.3 ± 1.1^a^
TG (mmol/L)	1.5 (1.1-2.2)	1.6 (1.1-2.3)	1.5 (1.1-2.1)
HDL-c (mmol/L)	1.1 (0.9-1.3)	1.1 (0.9-1.3)	1.0 (0.9-1.3)
LDL-c (mmol/L)	3.0 ± 0.9	3.1 ± 0.8	3.0 ± 1.0
eGFR (mL/min/1.73 m^2^)	89.0 ± 23.6	92.2 ± 24.9	87.8 ± 23.0
CRP (mg/L)	1.2 (0.6-3.1)	1.4 (0.5-3.1)	1.2 (0.6-2.9)
HOMA-IR	4.0 (2.8-5.9)	3.9 (2.8-5.9)	4.0 (2.8-6.1)
CSI	6 (2-12)	0 (0-1)	9 (4-14)^aa^
Smoking, n (%)	121 (44.5)	29 (37.2)	92 (47.4)
Diabetes ratio, n (%)	110 (40.4)	24 (30.8)	86 (44.3)^a^
Family history of CAD, n (%)	116 (42.6)	38 (48.7)	78 (40.2)
Hypoglycemic therapy, n (%)	62 (22.8)	11 (14.1)	51 (26.3)^a^
Anti-hypertensive therapy, n (%)	185 (68.0)	51 (65.4)	134 (69.1)
Lipid-lowering therapy, n (%)	76 (27.9)	11 (14.1)	65 (33.5)^aa^

CAD: Coronary artery disease; BMI: Body mass index; W: Waist circumference; SBP: Systolic blood pressure; DBP: Diastolic blood pressure; FPG: Fasting plasma glucose; 2hPG: 2-hour postprandial glucose; HbA1c: Glycated hemoglobin A1c; GA: Glycated albumin; 1,5-AG: 1,5-anhydroglucitol; TC: Total cholesterol; TG: Triglyceride; HDL-c: High density lipoprotein cholesterol; LDL-c: Low density lipoprotein cholesterol; eGFR: Estimated glomerular filtration rate; CRP: C-reactive protein; HOMA-IR: Homeostasis model assessment-insulin resistance index; CSI: Coronary stenosis index.

For the subjects: ^a^ *P* < 0.05, CAD vs. non-CAD; ^aa^ *P* < 0.01, CAD vs. non-CAD. Data are presented as mean ± standard deviation or median (interquartile range).

### Correlation analysis of glycemic markers

Spearman correlation analysis (Table 2) revealed that 1,5-AG was inversely related to FPG, 2hPG, HbA1c, and GA (*r* = –0.417, –0.482, –0.504, and –0.421, respectively; all *P* < 0.001), whereas GA was positively associated with FPG, 2hPG, and HbA1c (*r* = 0.469, 0.591, and 0.619, respectively; all *P* < 0.001). The relationships between glycemic markers (FPG, 2hPG, HbA1c, GA, and 1,5-AG) still existed in the partial correlation analysis after adjustment for age and gender (all *P* < 0.001).

**Table 2:** **Correlations of nontraditional (GA and 1,5-AG) and standard (FPG, 2hPG, and HbA1c) glycemic markers**

**Variables**	**HbA1c**		**GA**		**1,5-AG**	
	***r***	***P***	***r***	***P***	***r***	***P***
Spearman						
GA	0.619	<0.001	-	-	-	-
1,5-AG	–0.504	<0.001	–0.421	<0.001	-	-
FPG	0.577	<0.001	0.469	<0.001	–0.417	<0.001
2hPG	0.609	<0.001	0.591	<0.001	–0.482	< 0.001
Adjust for age and gender						
GA	0.709	< 0.001	-	-	-	-
1,5-AG	–0.579	< 0.001	–0.441	< 0.001	-	-
FPG	0.752	< 0.001	0.609	< 0.001	–0.495	< 0.001
2hPG	0.697	< 0.001	0.646	< 0.001	–0.525	< 0.001

### Multivariate analysis of factors contributing to CAD

For multivariate logistic regression analysis, the presence of CAD was a dependent variable; HbA1c, 1,5-AG, and GA were separate independent variables. After adjustment for traditional risk factors for CAD [gender, age, BMI, W, systolic blood pressure (SBP), diastolic blood pressure (DBP), TC, TG, HDL-c, LDL-c, eGFR, CRP, HOMA-IR, FPG, 2hPG, smoking status, family history of CAD, hypoglycemic therapy, anti-hypertensive therapy, and lipid-lowering therapy], the results showed that HbA1c [odds ratio (OR) = 1.343, 95% confidence interval (CI): 1.007-1.791, *P* = 0.044], 1,5-AG (OR = 0.957, 95% CI: 0.927-0.989, *P* = 0.008), and GA (OR = 1.143, 95% CI: 1.048-1.247, *P* = 0.002) were all the factors contributing to CAD.

Next, we created two models with the presence of CAD as the dependent variable to identify which indicator was most closely associated with CAD (Table 3). The independent variables included the traditional risk factors for CAD and the glycemic markers (HbA1c, 1,5-AG, and GA). The difference between the two models was the selection of glycemic markers. Model 1 only analyzed HbA1c and 1,5-AG and revealed that in addition to gender, age, and lipid-lowering therapy, 1,5-AG was independently associated with CAD (OR = 0.957, 95% CI: 0.927-0.989, *P* = 0.008). However, when HbA1c, 1,5-AG, and GA were simultaneously condensed into the equation in Model 2, GA remained as an independent risk factor for CAD (OR = 1.143, 95% CI: 1.048-1.247, *P* = 0.002).

**Table 3:** **Multivariate logistic regression analysis of independent factors associated with CAD**

**Independent variable**	***β***	**S.E.**	***P***	**OR**	**95% CI**
Model 1					
Gender (women)	–1.211	0.339	< 0.001	0.298	0.153-0.579
Age	0.055	0.016	0.001	1.057	1.023-1.092
Lipid-lowering therapy	1.223	0.401	0.002	3.396	1.548-7.452
1,5-AG	–0.044	0.017	0.008	0.957	0.927-0.989
Model 2					
Gender (women)	–1.101	0.334	0.001	0.332	0.173-0.640
Age	0.056	0.017	0.001	1.058	1.024-1.092
Lipid-lowering therapy	1.246	0.401	0.002	3.476	1.584-7.627
GA	0.134	0.044	0.002	1.143	1.048-1.247

Model 1: traditional risk factors for CAD (gender, age, BMI, W, SBP, DBP, TC, TG, HDL-c, LDL-c, eGFR, CRP, HOMA-IR, FPG, 2hPG, smoking status, family history of CAD, hypoglycemic therapy, anti-hypertensive therapy, lipid-lowering therapy), HbA1c and 1,5-AG.

Model 2: traditional risk factors for CAD, HbA1c, 1,5-AG and GA.

### Factors influencing the severity of coronary artery stenosis

Spearman correlation analysis showed that the CSI was positively associated with 2hPG, HbA1c, and GA (*r* = 0.197, 0.259, and 0.258, respectively; all *P* < 0.01) and inversely associated with 1,5-AG (*r* = –0.155, *P* = 0.010). No relationship was found between the CSI and FPG (*r* = 0.104, *P* = 0.086). After adjustment for gender and age, the CSI remained correlated with these glycemic markers above (all *P* < 0.05) and even showed a positive association with FPG (*r* = 0.135, *P* = 0.026).

A multiple stepwise regression model was used to analyze the independent factors affecting the severity of coronary artery stenosis (Table 4). The dependent variable was the CSI, and the independent variables were gender, age, BMI, W, SBP, DBP, TC, TG, HDL-c, LDL-c, eGFR, CRP, HOMA-IR, FPG, 2hPG, HbA1c, GA, 1,5-AG, smoking status, family history of CAD, hypoglycemic therapy, anti-hypertensive therapy, and lipid-lowering therapy. The results indicated that beyond gender, age, and lipid-lowering therapy, GA (standardized *β* = 0.184, *P* = 0.003) was independently correlated with the CSI.

**Table 4:** **Multiple stepwise regression analysis of CSI**

**Independent variable**	**Standardized** ***β***	***t*** **value**	***P***
Gender (women)	–0.264	–4.349	< 0.001
Age	0.194	3.182	0.002
Lipid-lowering therapy	0.245	4.046	< 0.001
GA	0.184	3.039	0.003

Variables of the original model included: gender, age, BMI, W, SBP, DBP, TC, TG, HDL-c, LDL-c, eGFR, CRP, HOMA-IR, FPG, 2hPG, HbA1c, GA, 1,5-AG, smoking status, family history of CAD, hypoglycemic therapy, anti-hypertensive therapy and lipid-lowering therapy.

## Discussion

HbA1c measurement is internationally recognized as the gold standard indicator of glycemic control, and this indicator is widely used to monitor glucose level in patients with diabetes. In recent years, the applications of HbA1c in the clinical setting have expanded to the screening, diagnosis, and prognosis of diabetes, making HbA1c a composite marker that combines a variety of functions [14]. In addition, HbA1c has also been demonstrated as an independent risk factor for cardiovascular disease [15]. However, HbA1c, influenced by hemoglobin update rate, drugs, and race, still bears some limitations since it can only reflect the mean blood glucose level over the previous 2 to 3 months. Unable to accurately reflect short-term glycemic variability, HbA1c has delayed effects when applied in adjustment for glucose-lowering treatments.

Some new indicators, such as GA and 1,5-AG, have emerged during recent years and provide HbA1c with complementary information. The study from Mukai, et al. [16] suggested the potential applicabilities of GA and 1,5-AG measurements as diagnostic tools for diabetes. GA, an early Amadori-type glycation protein of the nonenzymatic glycation reaction between glucose and serum albumin, is an index reflecting the average glucose level over the previous 2 to 3 weeks. Previous reports have demonstrated its advantage over HbA1c in reflecting glycemic excursion [17]. In addition, GA is strongly associated with incident diabetes and its microvascular complications, with prognostic value comparable to HbA1c [18]. But several factors, including the half-life of serum albumin, disorders such as thyroid disease and nephrotic syndrome, have been identified to exert effects on GA. 1,5-AG is the C1 deoxy form of glucofuranose and reflects the average blood glucose level during the past 1 to 2 weeks. This indicator is reportedly superior to HbA1c as a sensitive marker of postprandial hyperglycemia when the glucose level is above the renal glucose threshold [19,20]. 1,5-AG is known to be maintained at a steady-state level in the circulation, but chronic liver disease, severe renal impairment, cystic fibrosis, and the history of gastrectomy are known to affect 1,5-AG; however, there is few systemic report on its relationship with other glycemic markers and diabetic complications.

In our study, comparisons were performed for the first time among the associations of HbA1c, GA, and 1,5-AG with CAD in a Chinese population with high risk of CAD. Significant differences in 2hPG, HbA1c, GA, and 1,5-AG, but not FPG, were noted between subjects with and without CAD. All glycemic markers were highly correlated with each other. With respect to the ability of these indicators to reflect the blood glucose level over a certain period of time (HbA1c, the previous 2 to 3 months; GA, the previous 2 to 3 weeks; and 1,5-AG, the previous 1 to 2 weeks), GA and 1,5-AG were more closely correlated with CAD than was HbA1c. Additionally, GA appeared to be independently associated with the severity of coronary artery stenosis according to the calculated CSI.

Persistent hyperglycemia, especially elevated postprandial or postchallenge glucose level, may add to the risks of micro- or macrovascular complications in patients with diabetes [21]. After prospective analyses, the DECODE and DECODA group found that 2hPG was superior to FPG as a predictor of deaths from all causes and cardiovascular disease [22,23]. Moreover, there is growing evidence supporting links between postprandial hyperglycemia and oxidative stress, carotid intima-media thickness as well as endothelial dysfunction, all of which are recognized as markers of cardiovascular disease [21,24]. Given these clinical associations, postprandial hyperglycemia is closely related to both the risk and prognosis of cardiovascular disease. Considering the close correlation between the postprandial glucose level and CAD, a sensitive indicator of postprandial hyperglycemia may be more conducive to predicting the risk of CAD. Many studies have suggested that both GA and 1,5-AG reflect postprandial hyperglycemia better than does HbA1c [17,25]. In line with amounts of previous research [3-6,26], the present study showed that GA and 1,5-AG were more closely related to CAD than HbA1c.

It is purportedly that the causes of the abnormal distribution of peripheral blood lymphocytes play an important role in the occurrence and development of atherosclerosis [27]. Dworacka, et al. [28] found a correlation between 1,5-AG and abnormal distributions of CD4^+^ and CD8^+^28^-^ lymphocyte subsets in peripheral blood, and this correlation was stronger than that of HbA1c and the fasting blood glucose level. Ohira, et al. [29] showed that variations of 1,5-AG in response to the change of diabetic treatment were associated with the cardio-ankle vascular index, a known indicator of angiosclerosis. Furthermore, an 11-year prospective study performed by Watanabe, et al. [30] revealed that 1,5-AG was an independent risk factor for cardiovascular disease in men. So did the present study observe the close relationship between 1,5-AG and CAD, which was consistent with the findings of previous studies [6].

In the present study, GA was pointed out to be superior to HbA1c and 1,5-AG when associated with CAD. As a glycemic marker, GA is used to evaluate glycemic control during the previous 2 to 3 weeks. Norimatsu, et al. [26] selected 244 Japanese patients to undergo coronary computed tomography angiography, finding that GA was superior to HbA1c as a predictor of the presence of CAD. Furusyo, et al. [31,32] selected 1575 subjects aged 26 to 78 years for their Kyushu and Okinawa Population Study, finding that GA was positively associated with the carotid intima-media thickness and served as a predictor of the presence of atherosclerosis. A study of 218 Korean subjects carried out by Song, et al. [33] suggested that GA was a more valuable index than HbA1c for predicting the progression of atherosclerosis in patients with type 2 diabetes mellitus. In a series of research with enrollment of Chinese patients with type 2 diabetes mellitus undergoing coronary angiography, GA was identified as an important predictor of the presence and severity of CAD and exhibited predictive value beyond HbA1c [3,4]. Moreover, the higher GA level of type 2 diabetic patients concomitant with stable angina and chronic total occlusion was reportedly associated with the presence of diminished coronary collateral flow. Regression analysis revealed that GA, but not HbA1c, was independently associated with low coronary collateralization [5].

As a predominant early Amadori-type glycation protein in the circulation of patients with diabetes, GA plays a pivotal role in the physiological mechanisms of diabetic atherosclerosis. The particular mechanisms can be explained as follows. GA is transferred into advanced glycation end products in high-glucose conditions, which is a critical factor responsible for diabetic macrovascular complications. In addition, the soluble receptor of advanced glycation end products is also demonstrated to be an independent marker of CAD [34]. GA causes destruction in endothelial cell function, leading to oxidative stress and an inflammatory reaction in the vessel walls along with the proliferation and migration of vascular smooth muscle cells. In this way, GA speeds up the development and progression of atherosclerosis and vascular complications of diabetes [32,35].

### Limitations

The present study has two main limitations. First, we only recruited subjects admitted to the Department of Cardiology to undergo coronary angiography, and all subjects had already been on intervention therapy because of existing risk factors for CAD. This affected the results to some extent. Whether our findings can be replicated in the general population requires further confirmation. Second, limited by the small sample size and cross-sectional design, this study was hard to find the acute sequence of the increase in the GA level and occurrence of CAD. Further large-scale prospective studies are needed to determine whether GA is a suitable indicator for identifying CAD.

## Conclusions

GA displayed a more closely correlation with CAD than did HbA1c and 1,5-AG in a Chinese population with high risk of CAD. The findings of this study suggested that the serum GA level was superior to HbA1c and 1,5-AG in identifying CAD and that its measurement in patients with high risk of CAD was helpful for early detection of cardiovascular disease. Not only identification of the underlying connection between GA and atherosclerosis, but also utility of GA as a monitoring indicator of glucose control in diabetes management requires further investigation.

## Abbreviations

BMI: Body mass index
CAD: Coronary artery disease
CRP: C-reactive protein
CSI: Coronary stenosis index
DBP: Diastolic blood pressure
eGFR: Estimated glomerular filtration rate
FINS: Fasting serum insulin
FPG: Fasting plasma glucose
GA: Glycated albumin
HbA1c: Glycated hemoglobin A1c
HDL-c: High-density lipoprotein cholesterol
HOMA-IR: Homeostasis model assessment-insulin resistance index
LDL-c: Low-density lipoprotein cholesterol
SBP: Systolic blood pressure
Scr: Serum creatinine
TC: Total cholesterol
TG: Triglyceride
W: Waist circumference
1,5-AG: 1,5-anhydroglucitol
2hPG: 2-hour postprandial glucose
